# Potency of probiotics *Bifidobacterium spp*. and *Lactobacillus casei* to improve growth performance and business analysis in organic laying hens

**DOI:** 10.14202/vetworld.2019.860-867

**Published:** 2019-06-21

**Authors:** Widya Paramita Lokapirnasari, Teguh Bagus Pribadi, Anam Al Arif, Soeharsono Soeharsono, Sri Hidanah, Nenny Harijani, Rifqy Najwan, Khoirul Huda, Hana Cipka Pramuda Wardhani, Nabil Fariz Noor Rahman, Andreas Berny Yulianto

**Affiliations:** 1 Department of Animal Husbandry, Faculty of Veterinary Medicine, Universitas Airlangga, Jl. Mulyorejo, Kampus C, Universitas Airlangga, Surabaya, Indonesia; 2 Halal Research Center, Universitas Airlangga, Jl. Mulyorejo, Kampus C, Universitas Airlangga, Surabaya, Indonesia; 3 Magister of Veterinary Agribusiness, Faculty of Veterinary Medicine, Universitas Airlangga, Indonesia; 4 Department of Veterinary Anatomy, Faculty of Veterinary Medicine, Universitas Airlangga, Indonesia; 5 Department of Veterinary Public Health, Faculty of Veterinary Medicine, Universitas Airlangga, Indonesia; 6 Sains Veteriner, Faculty of Veterinary Medicine, Universitas Airlangga, Surabaya, Indonesia

**Keywords:** antibiotic growth promoters, business analysis, growth performance, hen-day production, probiotics

## Abstract

**Aim:**

This study aimed to determine the use of probiotics *Bifidobacterium* spp. and *Lactobacillus casei* as alternative antibiotic growth promoters (AGPs) to improve growth performance and business analysis.

**Materials and Methods:**

This study used a completely randomized factorial design. The first factor was the time of administration (1, 2, 3, and 4 weeks) and the second was the use of probiotics (control without probiotics; 0.1% AGP and 0.5% *Bifidobacterium* spp. + 0.25% *L. casei*). One hundred and eighty laying hens (Lohmann strain), of 30 weeks old, were divided into 12 treatment groups, composed of five replicates, each consisting of three laying hens.

**Results:**

The results showed that using 0.5% *Bifidobacterium* spp. + 0.25% *L. casei* in weeks 1 and 2 showed the lowest feed intake (FI) (112.11-112.19 g/day), the highest egg weight (60.28 g) in the 1^st^ week, the lowest feed conversion ratio (FCR) (2.21-2.23), and highest feed efficiency (44.75-45.25%) for 3-4 weeks, and the highest hen-day production (86.66-86.90%) for 3-4 weeks and the most profitable business analysis (IDR. 30,353).

**Conclusions:**

Based on the results, it can be concluded that the addition of 0.5% *Bifidobacterium* spp. + 25% *L. casei* probiotics can be used as a substitute for AGP; it can reduce the FI and FCR, increasing egg weight, feed efficiency, and hen-day production, as well as illustrating the results of the most profitable business analysis.

## Introduction

The use of antibiotics as growth promoters in poultry farms has been banned due to a myriad of reasons. In organic farms across Europe, the use of any drug, even for prevention or therapeutic purposes, is strictly monitored by the EC Council Regulation No. 1804/99 [1]. In Indonesia, it is overseen through the Minister of Agriculture Regulation No. 14 of 2017 concerning the Classification of Animal Medicine. The regulation is one of the government’s strategic steps in controlling the threat of antimicrobial resistance [2]. The limitation of antibiotic use has triggered a lot of research in an effort to look for other additives that could be used as an alternative for antibiotic growth promoters (AGPs). Probiotics is a feed additive that replaces the function of AGP in livestock includes probiotics.

Probiotics are live microbial cell preparations in the form of bacterial strains (generally lactic acid bacteria or Streptococcus) or yeast, which is given orally, alone or through a feed [3]. The use of probiotic supplementations containing beneficial bacteria, such as *Lactobacillus* spp. and *Bifidobacterium* spp. has a positive effect on the intestinal microbial population. Several researchers classify *Bifidobacterium* spp. and *Lactobacillus casei* as lactic acid bacteria categorized under probiotic microbial groups living in the digestive tract to improve its condition. The probiotic dose given through the poultry feed or drinking water is 0.1%-0.15% [4] or a 2% dose for more optimal results [5]. Giving probiotics to poultry could improve the feed conversion ratio (FCR), feed intake (FI), increase egg production, and stimulate growth rate as a result of hypocholesterolemic effects. The use of probiotics of different doses has been tested profusely, although an accurate dosage of administration has yet to be established [6].

Research on the use of probiotics as an alternative AGP is still very limited in obtaining high production results, impacting the economy of the livestock industry. This study aimed to determine the use of probiotics *Bifidobacterium* spp. and *Lactobacillus casei* as AGPs to improve growth performance and business analysis.

## Materials and Methods

### Ethical approval

An approval from the Institutional Ethical Committee (Faculty of Veterinary Medicine, Universitas Airlangga) was obtained before the experimental trial.

### Experimental design

This research used a completely randomized factorial design pattern. The first factor was the time of administration at intervals of 1, 2, 3, and 4 weeks, and the second was the use of probiotics: Control without probiotics, 0.1% AGP and 0.5% *Bifidobacterium* spp. + 0.25% *L. casei*. One hundred and eighty laying hens (Lohmann) of 30 weeks old were divided into 4×3 treatments, each group consisting of five replications made up of three. The dose of probiotics used in this study was based on the best doses that have been done in previous studies [7,8].

### Rearing system

Chicken placed in an individual battery cage (20 cm×35 cm×35cm). Nutrient content of laying period was as follows: Dry matter: 91.97%, ash: 9.28%, crude protein: 20.71%, extract ether: 6.36%, crude fiber: 7.43%; nitrogen-free extract: 48.18%, and energy metabolism (EM: 2938.60 Mcal/kg). The climatic conditions and lighting programs were operated and followed by the recommendations for the end of the experiment (±28-32°C; length lighting; 16 h/day; humidity; 55-65%).

To administer AGP, 0.1% AGP was mixed into the feed and evenly stirred. To administer the probiotics, 0.5% *Bifidobacterium* spp. + 0.25% *L. casei* (each concentration of 1.2×10^8^ colony forming units [CFU]/ml) was mixed with 7.5 ml/L of water (free of chlorine and other disinfectants). The probiotic solution was evenly sprayed on to a 1 kg feed ration, which was then dried for feeding. Probiotic solutions were freshly sprayed on the rations every day. Provision of feed rations in chickens was carried out every morning at 08.00 am and every noon at 1.00 pm. Drinking places were cleaned every day, while cage disinfection was conducted twice a month.

Diet composition was appropriate for the growing period of the hens and met the Standard National Indonesia requirements. Unconsumed feed was weighed and discarded weekly, and feed consumption data were carried out every week during the 4 weeks of treatment.

The analysis of growth performance was calculated using:

Feed intake (g) = Amount of feed given (g)−Amount of unconsumed feed;

FCR = Feed intake/Egg production;

Feed efficiency = (Egg production/Feed intake) × 100;

Hen-day production = Number of eggs per day×100.

The business analysis consisted of calculating the variable costs, receipts, and benefits of the control treatment (b0), use of 0.1% AGP (b1), and 0.5% *Bifidobacterium* spp. + 0.25% *L. casei* (b2) for 4 weeks of treatment.

The following formula was designed:

Total revenue = Price per unit×Total production;

Total cost = Total fixed cost+Total variable cost;

Profit = Total revenue - Total cost.

### Statistical analysis

Data were analyzed statistically using a univariate general linear model statistics to determine whether there were any interaction and significant differences between treatments. If the results were significantly different, then it was processed with the Duncan’s Multiple Distance Test. Data were then analyzed using the SPSS (IBM Corporation, USA) for Windows 22.0 program.

## Results

### FI

In the first factor, the difference in the length of time the additive feed is given shows a significant difference (p<0.05) to the FI. The lowest FI s are shown in administration for 1 week and 2 weeks, while the higher FIs are shown in administration for 3 and 4 weeks. In the second factor, giving a variety of feed additive showed a significant difference (p<0.05) between the treatment of the FI. The lowest FI was found with 0.5% *Bifidobacterium* spp. + 0.25% *L. casei*, followed by AGP. The highest FI was found in the control treatment. The results of this study indicate that there is an interaction (p<0.05) between the differences in the length of time given feed additive and the type of feed additive to FI in laying hens. The measurements of an average of FI in the treatment are listed in Table-1.

**Table-1:** Average of feed intake in the treatment (gram/hen/day).

Week factor	Feed additive factor			Average feed intake

	Control (b0)	0.1% AGP (b1)	0.5% *Bifidobacterium* spp. + 0.25% *L. casei* (b2)	
1 (a0)	115.14±0.87	113.04±0.73	112.11±0.78	113.43^a^±1.55
2 (a1)	115.32±0.50	113.18±0.40	112.19±0.58	113.56^a^±1.60
3 (a2)	116.34±0.76	114.47±1.08	113.42±1.35	114.74^b^±1.48
4 (a3)	116.35±0.42	114.62±0.71	113.60±0.75	114.86^b^±1.39
Average feed intake	115.79^c^±0.65	113.83^b^±0.83	112.83^a^±0.79	
Interactions a×b	a0×b0	a0×b1	a0×b2	
	115.14^c^±0.87	113.04^b^±0.73	112.11^a^±0.78	
	a1×b0	a1×b1	a1×b2	
	115.14^c^±0.51	113.04^b^±0.41	112.11^a^±0.59	
	a2×b0	a2×b1	a2×b2	
	116.34^b^±0.76	114.47^a^±1.09	113.42^a^±1.36	
	a3×b0	a3×b1	a3×b2	
	116.35^c^±0.42	114.62^b^±0.71	113.60^a^±0.75	

a,b,c Different superscripts in the same raw show significant difference (p<0.05). AGP=Antibiotic growth promoters

### Egg weight

There was a correlation between the length of time given by the factor feed additive both the use of AGP or 0.5% *Bifidobacterium* spp. + 0.25% *L. casei* and the weight of the eggs in laying hens. The highest egg weight is shown in administration for 1 week and the highest egg weight was found in 0.5% *Bifidobacterium* spp. + 0.25% *L. casei*, which was not different from the egg weight at 0.1% AGP. The lowest egg weight was in control without AGP and probiotics, and there was no significant difference found when compared to the administration for the second and fourth. The 0.5% *Bifidobacterium* spp. + 0.25% *L. casei* in the 2^nd^, 3^rd^, and 4^th^ weeks showed no difference compared to the use of 0.1% AGP in the 2^nd^ and 3^rd^ weeks. This shows that giving 0.5% *Bifidobacterium* spp. + 0.25% *L. casei* and 0.1% AGP provide relatively similar results. The measurements of egg weight during the 4 weeks of AGP and probiotic administration are listed in Table-2.

**Table-2:** Average of egg weight in the treatment (gram/egg).

Week factor	Feed additive factor			Average egg weight

	Control (b0)	0.1% AGP (b1)	0.5% *Bifidobacterium* spp. + 0.25% *L. casei* (b2)	
1 (a0)	60.00±0.81	59.71±0.48	60.28±0.75	60.00^a^±0.29
2 (a1)	58.71±0.48	59.28±0.48	58.57±0.53	58.85^c^±0.38
3 (a2)	58.42±0.78	59.00±0.81	58.57±0.78	58.66^c^±0.30
4 (a3)	58.71±0.48	60.00±0.81	59.14±0.69	59.28^b^±0.66
Average egg weight	58.96^a^±0.71	59.50^b^±0.44	59.14^ab^±0.81	
Interactions a×b	a0×b0	a0×b1	a0×b2	
	60.00^a^±0.82	59.71^a^±0.49	60.28^a^±0.76	
	a1×b0	a1×b1	a1×b2	
	60.00^a^±0.49	59.71^a^±0.49	60.28^a^±0.53	
	a2×b0	a2×b1	a2×b2	
	58.42^a^±0.79	59.00^a^±0.82	58.57^a^±0.79	
	a3×b0	a3×b1	a3×b2×b2	
	58.71^a^±0.49	60.00^b^±0.82	59.14^a^±0.69	

a,b,c Different superscripts in the same raw show significant difference (p<0.05). AGP=Antibiotic growth promoters, *L. casei*=*Lactobacillus casei*

### FCR and feed efficiency

In the first factor, the difference in the length of time the additive feed was given shows a significant difference (p<0.05) to the FCR and feed efficiency. The lowest FCR was shown in administration for 1 week, 3 and 4 weeks, while the higher FCR was shown in administration for 2 weeks. In the second factor, giving a variety of feed additive showed a significant difference (p<0.05) between the treatment of the FCR. The lowest FCR was found with 0.5% *Bifidobacterium* spp. + 0.25% *L. casei* followed by AGP. The highest FCR was found in the control treatment. There was an interaction (p<0.05) between the length of time given feed additive and the type of feed additive to FCR in laying hens. Data from the research on FCR are listed in Table-3. The best conversion ratio (2.21-2.45) was produced at 0.5% *Bifidobacterium* spp. + 0.25% *L. casei* for 1-4 weeks administration. The results of the average FCR are listed in Table-3.

**Table-3:** Average of FCR in the treatment.

Week factor	Feed additive factor			Average FCR

	Control (b0)	0.1% AGP (b1)	0.5% *Bifidobacterium* spp. + 0.25% *L. casei* (b2)	
1 (a0)	2.54±0.04	2.54±0.04	2.45±0.08	2.53^c^±0.05
2 (a1)	2.61±0.02	2.46±0.06	2.32±0.04	2.46^b^±0.15
3 (a2)	2.63±0.02	2.40±0.03	2.23±0.03	2.41^a^±0.20
4 (a3)	2.62±0.04	2.41±0.06	2.21±0.05	2.41^a^±0.21
Average FCR	2.61^c^±0.04	2.45^b^±0.06	2.30^a^±0.11	
Interactions a×b	a0×b0	a0×b1	a0×b2	
	2.62^c^±0.04	2.54^b^±0.04	2.45^a^±0.08	
	a1×b0	a1×b1	a1×b2	
	2.62^c^±0.03	2.54^b^±0.07	2.45^a^±0.05	
	a2×b0	a2×b1	a2×b2	
	2.60^c^±0.02	2.40^b^±0.04	2.23^a^±0.03	
	a3×b0	a3×b1	a3×b2	
	2.61^c^±0.05	2.41^b^±0.06	2.21^a^±0.06	

a,b,c Different superscripts in the same raw show significant difference (p<0.05). FCR=Feed conversion ratio, *L. casei*=*Lactobacillus casei*

There was an interaction between the length of time given by the feed additive factor and the use of AGP or 0.5% *Bifidobacterium* spp. + 0.25% *L. casei* in feed efficiency of laying hens. The highest feed efficiency results were indicated by giving 0.5% *Bifidobacterium* spp. + 0.25% *L. casei* for 3-4 weeks. The lowest feed efficiency was found in the control where AGP and probiotics were not given, both in the 1^st^ week and the 4^th^ week of treatment. The results of the average feed efficiency are listed in Table-4.

**Table-4:** Average of feed efficiency in the treatment.

Week factor	Feed additive factor			Average feed efficiency

	Control (b0)	0.1% AGP (b1)	0.5% *Bifidobacterium* spp. + 0.25% *L. casei* (b2)	
1 (a0)	38.08±0.60	39.36±0.65	40.85±1.38	39.43^a^±1.39
2 (a1)	38.05±0.37	40.66±1.15	43.12±0.84	40.61^b^±2.54
3 (a2)	38.37±0.32	41.60±0.70	44.75±0.64	41.57^c^±3.19
4 (a3)	38.32±0.69	41.50±1.07	45.25±1.19	41.69^c^±3.47
Average feed efficiency	38.21^a^±0.16	40.78^b^±1.04	43.49^c^±1.98	
Interactions a×b	a0×b0	a0×b1	a0×b2	
	38.08^a^±0.61	39.36^b^±0.66	40.85^c^±1.39	
	a1×b0	a1×b1	a1×b2	
	38.08^a^±0.38	39.36^b^±1.15	40.85^c^±0.84	
	a2×b0	a2×b1	a2×b2	
	38.37^a^±0.32	41.60^b^±0.71	44.75^c^±0.65	
	a3×b0	a3×b1	a3×b2	
	38.32^a^±0.70	41.50^b^±1.08	45.25^c^±1.19	

a,b,c Different superscripts in the same raw show significant difference (p<0.05). AGP=Antibiotic growth promoters, *L. casei*=*Lactobacillus casei*

### Hen-day production

In the first factor, the difference in the length of time the additive feed was given shows a significant difference (p<0.05) to the hen-day production. The highest HDP were shown in administration for 3 weeks and 4 weeks, while the lower HDP were shown in administration for 2 and 1 weeks, respectively. In the second factor, giving a variety of feed additive showed a significant difference (p<0.05) between the treatment of the HDP. The highest HDP was found with 0.5% *Bifidobacterium* spp. + 0.25% *L. casei* followed by AGP. The lowest HDP was found in the control treatment. A correlation was identified between the length of time given by the factor feed additive with the use of either AGP or 0.5% *Bifidobacterium* spp. + 0.25% *L. casei* to hen-day production in laying hens. The highest hen-day production was shown by giving 0.5% *Bifidobacterium* spp. + 0.25% *L. casei* for 3-4 weeks followed by 2 and 1 week. Data from the research on HDP care shown in Table-5.

**Table-5:** Average of hen-day production in the treatment.

Week factor	Feed additive factor			Average HDP

	Control (b0)	0.1% AGP (b1)	0.5% *Bifidobacterium* spp. + 0.25% *L. casei* (b2)	
1 (a0)	73.09±1.49	74.52±1.26	75.95±1.31	74.52^a^±1.43
2 (a1)	74.76±1.50	77.62±1.62	82.61±1.88	78.33^b^±3.97
3 (a2)	76.43±1.50	80.71±0.89	86.66±1.66	81.27^c^±5.14
4 (a3)	75.95±0.89	79.28±1.62	86.90±1.49	80.71^c^±5.61
Average HDP	75.06^a^±1.49	78.03^b^±2.66	83.03^c^±5.11	
Interactions a×b	a0×b0	a0×b1	a0×b2	
	73.09^a^±1.50	74.52^ab^±1.26	75.95^b^±1.31	
	a1×b0	a1×b1	a1×b2	
	73.09^a^±1.50	74.52^ab^±1.62	75.95^b^±1.89	
	a2×b0	a2×b1	a2×b2	
	76.43^a^±1.50	80.71^b^±0.89	86.66^c^±1.67	
	a3×b0	a3×b1	a3×b2	
	75.95^a^±0.89	79.28^b^±1.63	86.90^c^±1.50	

a,b,c Different superscripts in the same raw show significant difference (p<0.05). AGP=Antibiotic growth promoters, *L. casei*=*Lactobacillus casei*

### Business analysis

The business analysis consists of calculating variable costs, revenues, and profits. The results of the calculation of the average variable costs, acceptance and control advantages, and the use of AGP and probiotics are listed in Table-6.

**Table-6:** The average variable costs, revenue, and profit of control, and the use of AGP and probiotics.

Treatment	Variable costs	Revenue	Profit
		
	(IDR/day)	(IDR/day)	(IDR/day)
Control (b0)	38.364.74^c^±198.01	61.082.50^c^±1502.84	22.717.79^c^±1694.16
0.1% AGP (b1)	37.719.01^b^±241.70	64.010.80^b^±1272.51	26.291.80^b^±1361.36
0.5% *Bifidobacterium* spp. + 0.25% *L. casei* (b2)	37.372.17^a^±213.46	67.725.68^a^±2039.11	30.353.52^a^±2012.40

a,b,c Different superscripts in the same raw show significant difference (p<0.05). AGP = Antibiotic growth promoter

## Discussion

### FI

FI is the amount of ration consumed by chickens over 24 h, calculated using the difference between the feed given and the feed left over. Based on the results in Table-1, there were significant differences (p<0.05) among treatments. The lowest FI was found in the use of a combination of 0.5% *Bifidobacterium* spp. + 0.25% *L. casei* for 1-4 weeks. The highest FI results were found in control without the use of feed additives in the 1, 2, and 4^th^ weeks. A high FI is a result of limited absorption of nutrients in the small intestine, therefore, that the feed ration consumed by chickens is greater. A previous study found that a good FI is shown by giving 0.5% *Bifidobacterium* spp. + 0.25% *L. casei* to layer chickens, showing similar results to the current study [9] using probiotics containing *Bifidobacterium* *thermophilum*, *Lactobacilli*, and *Enterococcus faecium*. Administering probiotics to chickens can increase jejunal villus height and reduce the villus-crypt depth. The use of *Lactobacillus acidophilus* has a positive role in poultry intestinal mucosa because it strengthens the barrier effect. However, the positive effects are dependent on the adhesion and replication on the intestinal wall [10] hence why the use of probiotics containing *B. subtilis, B. subtilis* C-3102 [11-13], *L. acidophilus* and a mixture of *Lactobacillus* spp., *B. cereus* in broiler chickens, can increase the concentration of volatile fatty acids in the ileum and cecum, as well as reduce the pH value. Decreased pH in the digestive tract is associated with a decline in the number of *E. coli* forms and an increase in bacteria which is beneficial for the intestines [14]. The decrease in the population of *E. coli* in the intestine is associated with an increase in the number of *Lactobacillus* spp. [10]. It supports the hypothesis that *Lactobacilli* could compete with *E. coli* for intestinal colonization. Watkins observed that a competitive exclusion against pathogenic *E. coli* strains occurred in gnotobiotic chicks that were fed *L. acidophilus*. Probiotics contain antagonistic abilities toward several pathogenic bacteria, such as *E. coli*, *Salmonella* spp., and *Shigella* spp. [15].

Administering combination cultures of *Lactobacillus* and *Bacillus* spp. in laying hens showed the results of increased feed consumption and drinking water [16]. Viable *Lactobacillus* at 1100 mg/kg (4.4×10^7^ CFU/kg) dramatically increased egg size, daily feed consumption, calcium retentions, and nitrogen [17]. Some of the results, however, did not show an increase in feed consumption [18], egg production, and egg weight [19]. There seemed to be no effect on FI, egg production, or egg mass of hens throughout the 48 weeks [20].

Feed intake and the energy balance in poultry, influenced by genetic regulation [21]. Growth and fat deposition are related to the level of FI and feed efficiency, which can be affected by several genes; those that are significantly related to the FI are LCORL (ligand-dependent nuclear receptor corepressor-like) and NCAPG (non-SMC condensin I complex, subunit G) genes [22]. Egg production and egg weight are also affected by the chromosome position [23]. The increase in FI in the administration of probiotics is due to an increase in digestion, absorption, and availability of nutrition, positively effecting intestine activity, and increasing digestive enzymes [24].

### Egg weight

There is an interaction between the length of time given by the feed additive with either the use of AGP or 0.5% *Bifidobacterium* spp. + 0.25% *L. casei* on egg weight in laying hens. Data from research results on Egg weight are presented in Table-5.

The addition of probiotics did not have a significant effect on egg production and mass, but it did affect egg weight [25]. The same result was reported that supplementation of *Lactobacillus* cultures did not influence the egg production of hens throughout the experimental period and no significant difference in egg weight in hens fed with *L. acidophilus* [20]. Other research could also not identify a significant improvement in egg production of hens supplemented with PrimaLac containing *Lactobacillus* species [26]. The highest egg weight was found in the recent study of 0.5% *Bifidobacterium* spp. + 0.25% *L. casei*, while the lowest egg weight was in control without the use of AGP or probiotics. The mechanism of given probiotic to laying hens in the present study could be due to a decrease of pathogenic bacteria proliferation resulting from a change in the environment of the gastrointestinal tract and enhanced nutrient utilization, and it could be due to increased enzymatic activity in the gut resulting in improving nutrient utilization [27]. Giving the probiotics could improve the structure of the intestinal mucosa, increased villus length of the intestinal mucosa implies that there would be an increased surface area for nutrient absorption [28].

### FCR and feed efficiency

The FCR is the result of a comparison between the FI (g) and egg production (g). The results showed that there were significant differences (p<0.05) between treatments, the lowest FCR being found in the treatment of 0.5% *Bifidobacterium* spp. + 0.25% *L. casei* was used for 1-4 weeks. The highest feed conversion results were found in control without the use of AGP or probiotics in week 1, 2, 3, and week 4. The giving of 0.5% *Bifidobacterium* spp. + 0.25% *L. casei* for 1-4 weeks showed FCR results that were significantly different from the administration of 0.1% AGP for weeks 1, 2, 3, and 4. High FCR results were found in controls without the giving of AGP and probiotics. High FCR results showed lower feed efficiency. The low FCR is caused by giving probiotics can reduce feed consumption, but the egg production produced is the highest. The decrease in FCR indicates an increase in feed efficiency ratio. Feed efficiency can be used to evaluate the efficacy of nutrient and energy metabolic processes. Increased feed efficiency has a positive impact because it can reduce production costs for producers. FCR is the main index to find out and assess feed efficiency in livestock because it affects the increase in growth performance. Feed efficiency is influenced by several factors, not only by physiological states and genetic hosts but also by the intestinal microbiota. Microbiota in the intestinal tract can affect nutrient digestion and energy absorption in the host. *Lactobacillus* is one of the most dominant microbiotas found in the duodenum in chickens, resulting in better feed efficiency [29]. This improvement in feed efficiency and reduction in FCR by additional probiotic could be related to its promoting effects on metabolism of digestion processes and utilization of nutrients [27]. The other study supplemented probiotic with 0.1% and 0.2% in laying hens (age 54 weeks) for 3 months reported an increase in egg production and decreases in FCR and FI [30]. The mechanism explained that the increased egg production by probiotic might be due to the elongated shape of small and large intestine as well as their suppressing effects on pathogen bacteria and stimulating effects on the growth and activity of beneficial bacteria probiotic in the intestines which increase absorption of nutrient [31].

The administration of *Lactobacillus* and *Bifidobacterium* in this study is consistent with Yan that *Lactobacillus* is strongly associated with feed efficiency in the host. *Lactobacillus* is a beneficial bacteria that are commensal, which is beneficial to humans and animals. It can be concluded that *Lactobacillus* enriched generally can improve the digestive tract and thus protect the intestine from pathogens and promote efficient nutrition and energy extraction in the host. Changes in the composition of the microbiota in the intestine will affect host metabolism [32,33].

### Hen-day production

There is an established relationship between the length of time given by the factor feed additive and either the use of AGP or 0.5% *Bifidobacterium* spp. + 0.25% *L. casei* to hen-day production in laying hens. Data from the research on hen-day production are shown in Table-5.

Hen-day production is a comparison of egg production and the number of chickens multiplied by 100. The results found significant differences (p<0.05) between treatments. The highest hen-day production is found in the treatment of a2b2 and a3b2, where 0.5% *Bifidobacterium* spp. + 0.25% *L. casei* is used in the 3^rd^ and 4^th^ weeks of HDP of 86.66% and 86.90%. The lowest hen-day production is found in the control for 1-4 weeks. The use of AGP shows lower hen-day production values of 14.24% compared to probiotic use.

The administration of probiotics containing *Bifidobacterium* and *L. casei* is in accordance with studies that report the use of liquid probiotic mixed culture probiotics containing two types of microorganisms, *Lactobacillus* and *Bacillus* species. These species showed the highest yield day production and egg weight in layers [16], as well as in laying hens given a mixed culture of *L. acidophilus*, *L. casei*, and *L. acidophilus* [34]. Those given probiotics showed significant improvement in egg production and that layers fed with probiotics contained *Bifidobacterium* *bifidum*, *Lactobacillus plantarum*, *L. acidophilus*, *Lactobacillus delbrueckii* subsp. *bulgaricus, Lactobacillus rhamnosus, Streptococcus salivarius* subsp. *thermophilus, Aspergillus oryzae*, *E. faecium*, and *Candida pintolopesii* showed greater egg production than the group fed with basal diet [30].

Control group shows the lowest HDP value as a result of the absorption of nutrients that are limited compared to the treatment with the use of AGP or probiotic feed additives. The AGP treatment showed higher egg production compared to control because the addition of AGP in the ration helped reduce the number of intestinal microflora and suppressed the growth of pathogenic bacteria in the digestive tract. However, the disadvantage of using AGP includes a residue from the antibiotics being detected in livestock products such as meat, eggs, and milk, which is dangerous for consumers. The combination of 0.5% *Bifidobacterium* spp. + 0.25% *L. casei* showed the highest egg production results. This is because probiotics can produce an acidic atmosphere in the digestive tract, therefore suppressing the growth of pathogenic bacteria. The condition of a good digestive tract can help improve the metabolic process and absorption of nutrients needed by the body. Provision of probiotics *L. casei* and *L. rhamnosum* can increase feed efficiency and egg production while reducing production costs [35]. The use of probiotics can improve egg production, eggshell weight, eggshell thickness, and reduce cholesterol levels in the egg yolk [36].

### Business analysis

Business analysis consists of calculating variable costs, receipts, and profits. The results of calculation over 4 weeks are listed in Table-6, highlighting significant differences (p<0.05) between treatments b0, b1, and b2.

### Variable costs

Variable costs are the number of marginal costs for all units produced. They are calculated by multiplying the cost of feed and the additives (AGP and Probiotics) by the number of livestock units. Table-6 shows that there are significant differences (p<0.05) between treatments b0, b1, and b2. The highest variable cost in treatment b0 is IDR. 38,364.74, because the average FI for 4 weeks of treatment without AGP or probiotics is equal to 115.79 g/head/day. In the treatment with 0.1% AGP (b1), a lower variable cost was produced IDR. 37,719.01, compared to b0. b1 had an average FI for 4 weeks treatment of 113.83 g/head/day. The lowest variable cost in the treatment of b2 is IDR. 37,372.17, with an average FI for 4 weeks treatment of 112.83 g/head/day.

### Revenue

Revenue is the selling price per unit of production multiplied by the number of products sold. It is determined by egg production, the higher it is, the higher the acceptance that will be received.

Table-6 shows that a significant difference (p<0.05) was found among treatments. The treatment of b0, with the provision of basal feed without probiotics or AGP, had the lowest egg sales revenue at IDR 61,082.50, with an HDP of 75.06%. Treatment of b1 shows lower results than P2 at IDR. 64,010.80, with an HDP of 78.03%. The highest treatment was b2, with egg sales totaling IDR. 67,725.68, with an HDP of 83.03%.

### Profit

Profits are obtained through total income minus production costs. To meet the conditions of profit, the income has to be greater than the cost of production. Based on Table-6 showed a significant difference (p<0.05) among treatments. Treatment b2 shows the largest profit of totaling IDR. 30,353.52, a result of the lowest variable costs and the highest revenues. Treatment b1 has a profit of Rp. 26,291.80, while b0 is the treatment with the lowest profit at IDR. 22,717.79, having the highest variable costs and the lowest receipts. The advantages of probiotic use (a combination of 0.5% *Bifidobacterium* spp. + 25% *L. casei*) include improving feed efficiency to reduce the overall cost, as well as increasing egg production. The lower costs of feed and higher production of eggs determine increased profits for farmers.

## Conclusion

Based on the results, it can be concluded that the addition of 0.5% *Bifidobacterium* spp. + 25% *L. casei* probiotics can be used as an alternative substitute for AGP. The use of probiotics can reduce the FI and FCR, therefore increasing egg weight, feed efficiency, and hen-day production, as well as illustrating the results of the most profitable business analysis.

## Authors’ Contributions

WPL designed, interpreted, and analyzed data in the research and drafted manuscript; SH, NH, SS, and ABY interpreted data; TBP, RN, KH, HCPW, and NFNR conducted the field experimental work and participated in data collection; and AA who analyzed and interpreted the data. All authors read and approved the final manuscript.

## References

[1] EC Council Regulation (1999). EC Council Regulation No. 1804/99 of July 1999 Supplementing Regulation (EEC) No. 2092/91 on Organic Production of Agricultural Products. Official Journal L. 222128.

[2] Permentan (2017). Regulation of the Minister of Agriculture Republic Indonesia Nomor14/PERMENTAN/PK.350/5/2017 about Classification of Animal Medicine.

[3] Kochewad S.A, Chahande J.M, Kanduri A.B, Deshmukh D.S, Ali S.A, Patil V.M (2009). Effect of probiotic supplementation on growth parameters of Osmanabadi kids. Vet. World.

[4] Rowghani E, Arab M, Akbarian A (2007). Effects of probiotic and other feed additives on performance and immune response of broiler chicks. Int. Poult. Sci.

[5] Shareef A.M, Al-Dabbagh A.S (2009). Effect probiotic (*Saccharomyces cerevisiae*) on performance of broiler chicks. Iraqi J. Vet. Sci.

[6] Getachew T (2016). A review on effects of probiotic supplementation in poultry performance and cholesterol levels of egg and meat. J. World Poult. Res.

[7] Lokapirnasari W.P, Rahmawati A, Eliyani H (2016). Potential addition of *Lactobacillus casei* and *Lactobacillus rhamnosus* lactic acid bacteria to feed consumption and feed conversion of broiler. J. Agro Vet.

[8] Lokapirnasari W.P, Sahidu A.M, Soepranianondo K, Supriyanto A, Yulianto A.B, Al Arif A (2018). Potency of lactic acid bacteria isolated from Balinese bovine (*Bos sondaicus*) intestinal waste from slaughterhouse to improve nutrient content of wheat pollard as animal feedstuff by fermentation process. Vet. World.

[9] Chichlowski M, Croom J, McBride B.W, Daniel L, Davis G, Koci M.D (2007). Direct-fed microbial primalac and salinomycin modulate whole-body and intestinal oxygen consumption and intestinal mucosal cytokine production in the broiler chick. Poult. Sci.

[10] Forte C, Acuti G, Manuali E, Proietti P.C, Pavone S, Trabalza-Marinucci M, Moscati L, Onofri A, Lorenzetti C, Franciosini M.P (2016). Effects of two different probiotics on microflora, morphology, and morphometry of gut in organic laying hens. Poult. Sci.

[11] Abdelqader A, Al-Fataftah A.R, Daş G (2013). Effects of dietary *Bacillus subtilis* and inulin supplementation on performance, eggshell quality, intestinal morphology and microflora composition of laying hens in the late phase of production. Anim. Feed Sci. Technol.

[12] Knap I, Kehlet A.B, Bennedsen M, Mathis G.F, Hofacre C.L, Lumpkins B.S, Jensen M.M, Raun M, Lay A (2011). *Bacillus subtilis*(DSM17299) significantly reduces *Salmonella* in broilers. Poult. Sci.

[13] Jeong J.S, Kim I.H (2014). Effect of *Bacillus subtilis* C-3102 spores as a probiotic feed supplement on growth performance, noxious gas emission, and intestinal microflora in broilers. Poult. Sci.

[14] Li S.P, Zhao X.J, Wang J.Y (2009). Synergy of *Astragalus* polysaccharides and probiotics (*Lactobacillus* and *Bacillus cereus*) on immunity and intestinal microbiota in chicks. Poult. Sci.

[15] Watkins B.A, Miller B.F, Neil D.H (1982). *In vivo* inhibitory effects of *Lactobacillus acidophilus* against pathogenic *Escherichia coli* in gnotobiotic chicks. Poult. Sci.

[16] Pambuka S.R, Sjofjan O, Radiati L.E (2014). Effect of liquid probiotics mixed culture supplements through drinking water on laying hens performance and yolk cholesterol. J. Worlds Poult. Res.

[17] Nahashon S.N, Nakaue H.S, Mirosh L.W (1996). Performance of single comb white leghorn fed a diet supplemented with a live microbial during the growth and egg laying phases. Anim. Feed Sci. Technol.

[18] Yousefi M, Karkoodi K (2007). Effect of probiotic thepax®and *Saccharomyces cerevisiae* supplementation on performance and egg quality of laying hens. Int. J. Poult. Sci.

[19] Mahdavi A.H, Rahman H.R, Pourreza J (2005). Effect of probiotic supplements on egg quality and laying hen's performance. Int. J. Poult. Sci.

[20] Ramasamy K, Abdullah N, Wong M.C, Karuthan C, Ho Y.W (2010). Bile salt deconjugation and cholesterol removal from media by *Lactobacillus* strains used as probiotics in chickens. J. Sci. Food Agric.

[21] Richards M.P (2003). Genetic regulation of feed intake and energy balance in poultry. Poult. Sci.

[22] Lindholm-Perry A.K, Sexten A.K, Kuehn L.A, Smith T.P, King D.A, Shackelford S.D, Wheeler T.L, Ferrell C.L, Jenkins T.G, Snelling W.M, Freetly H.C (2011). Association, effects and validation of polymorphisms within the NCAPG-LCORL locus located on BTA6 with feed intake, gain, meat and carcass traits in beef cattle. BMC Genet.

[23] Wolc A, Arango J, Settar P, Fulton J.E, O'sullivan N.P, Preisinger R, Habier D, Fernando R, Garrick D.J, Hill W.G, Dekkers J.C.M (2012). Genome-wide association analysis and genetic architecture of egg weight and egg uniformity in layer chickens. Anim. Genet.

[24] Edens F.W (2003). An alternative for antibiotic Se in poultry *Probiotics*. Rev. Bras. Ciênc. Avíc.

[25] Daneshyar M, Kermanshahi H, Golian A (2009). Changes of biochemical parameters and enzyme activities in broiler chickens with cold-induced ascites. Poult. Sci.

[26] Davis G.S, Anderson K.E (2002). The effects of feeding the direct-fed microbial, primalac, on growth parameters and egg production in single white leghorn hens. Poult. Sci.

[27] Khan S.H, Atif M, Mukhtar N, Rehman A, Fareed G (2011). Effects of supplementation of multi-enzyme and multi-species probiotic on production performance, egg quality, cholesterol level and immune system in laying hens. J. Appl. Anim. Res.

[28] Inatomi T (2016). Laying performance, immunity and digestive health of layer chickens fed diets containing a combination of three probiotics. Sci. Postprint.

[29] Yan W, Sun C, Yuan J, Yang N (2017). Gut metagenomic analysis reveals prominent roles of *Lactobacillus* and cecal microbiota in chicken feed efficiency. Sci. Rep.

[30] Yörük M.A, Gül M, Hayirli A, Macit M (2004). The effects of supplementation of humate and probiotic on egg production and quality parameters during the late laying period in hens. Poult. Sci.

[31] Chen Y.C, Nakthong C, Chen T.C (2005). Improvement of laying hen performance by dietary prebiotic chicory oligofructose and inulin. Int. J. Poult. Sci.

[32] Stanley D, Geier M.S, Denman S.E, Haring V.R, Crowley T.M, Hughes R.J, Moore R.J (2013). Identification of chicken intestinal microbiota correlated with the efficiency of energy extraction from feed. Vet. Microbiol.

[33] Turnbaugh P.J, Bäckhed F, Fulton L, Gordon J.I (2008). Diet-induced obesity is linked to marked but reversible alterations in the mouse distal gut microbiome. Cell Host Microbe.

[34] Haddadin M.S.Y, Abdulrahim S.M, Hashlamoun E.A.R, Robinson R.K (1996). The effect of *Lactobacillus acidophilus* on the production and chemical composition of hen's eggs. Poult. Sci.

[35] Lokapirnasari W.P, Dewi A.R, Fathinah A, Hidanah S, Harijani N, Soeharsono S, Karimah B, Andriani A.D (2017). Effect of probiotic supplementation on organic feed to alternative antibiotic growth promoter on production performance and economics analysis of quail. Vet. World.

[36] Panda A.K, Reddy M.R, Rao S.V.R, Praharaj N.K (2003). Production performance, serum/yolk cholesterol and immune competence of white leghorn layers as influenced by dietary supplementation with probiotic. Trop. Anim. Health Prod.

